# Caveolin-1 mitigates the advancement of metabolic dysfunction-associated steatotic liver disease by reducing endoplasmic reticulum stress and pyroptosis through the restoration of cholesterol homeostasis

**DOI:** 10.7150/ijbs.100794

**Published:** 2025-01-01

**Authors:** Hanlin Xu, Yu Li, Ning Guo, Shuai Wu, Can Liu, Zhongxuan Gui, Weiju Xue, Xiangfu Jiang, Mengjia Ye, Qianqian Geng, Xiaowen Feng, Chao Zhang, Lei Jin, Chengmu Hu

**Affiliations:** 1 Inflammation and Immune Mediated Diseases Laboratory of Anhui Province, Anhui Institute of Innovative Drugs, School of Pharmacy, Anhui Medical University, Hefei, China;; 2 Institute for Liver Diseases of Anhui Medical University, School of Pharmacy, Anhui Medical University, Hefei, China.; 3 Key Laboratory of Anti-inflammatory and Immune Medicine, Ministry of Education, Hefei, China.; 4 Department of Cardiovascular Surgery, The First Affiliated Hospital of Anhui Medical University, Hefei, China.; 5 Oncology Department of Integrated Traditional Chinese and Western Medicine, The First Affiliated Hospital of Anhui Medical University, Hefei, China.; 6 Department of Pharmacy, The Second People's Hospital of Hefei, Hefei Hospital Affiliated to Anhui Medical University, Hefei, Anhui, China.; 7 Department of Hepatobiliary and Pancreatic Surgery, The First Affiliated Hospital of Anhui Medical University, Hefei, China.; 8 Department of Infectious diseases, The Second Affiliated Hospital of Anhui Medical University, China.

**Keywords:** Caveolin-1, MASLD, Cholesterol, ER tress, Pyroptosis

## Abstract

Metabolic dysfunction-associated steatotic liver disease (MASLD) is the most prevalent chronic liver disease worldwide, which has the potential to advance to fibrosis. CAV1 has the effects of improving liver lipid deposition in MASLD, however, the potential mechanism is largely unknown. Here, we establish a MASLD mouse model in CAV1 knockout (KO) mice and perform transcriptome analysis on livers from mice to investigate the effects of CAV1 in MASLD progression. In addition, we evaluated the expression of CAV1 in human liver samples, and also conducted assays *in vitro* to investigate the molecular role of CAV1 in MASLD progression. The results illustrate that the expression of liver CAV1 in the decreases during MASLD progression, which aggravates the accumulation of cholesterol in the liver, leading to more severe endoplasmic reticulum (ER) stress and pyroptosis. Mechanistically, CAV1 regulates the expression of FXR/NR1H4 and its downstream cholesterol transporter, ABCG5/ABCG8, suppressing ER stress and alleviating pyroptosis. Our study confirms CAV1 is a crucial regulator of cholesterol homeostasis in MASLD and plays an important role in disease progression.

## Introduction

Metabolic dysfunction-associated steatotic liver disease (MASLD) is a hepatic manifestation of metabolic syndrome, which is characterized by excessive fat accumulation, without the presence of long-term alcohol consumption 1, with current estimates suggesting that it affects 38% of the world population 2. Metabolic dysfunction-associated steatohepatitis (MASH), the progressive form of MASLD, characterized by hepatic steatosis and inflammation, hepatocyte ballooning, and the potential to advance to liver fibrosis, cirrhosis, and HCC 3.

Excessive accumulation of fat and lipotoxic metabolites in liver cells is widely recognized as a crucial factor in the development of MASLD, the proinflammatory response in the liver is closely associated with progression from MASH to fibrosis 4. Recent evidence has shown that some lipotoxic lipids, such as free cholesterol (FC), accumulation induces hepatocyte death and subsequent inflammation and fibrosis in the pathogenesis of MASH 5. However, the specific lipotoxic molecules involved have not yet been identified. Lipotoxic molecules, such as FC and its derivatives, accumulation in hepatic mitochondria elicit mitochondrial dysfunction and activates the unfolded protein response in the endoplasmic reticulum (ER), resulting in ER stress and hepatocyte apoptosis 7. Recent experimental and clinical findings have suggested that MASLD development involves alterations in hepatic cholesterol homeostasis and FC accumulation 7. Cholesterol-mediated inflammatory transitions in the liver affect the pathogenesis of MASLD and lead to pathological consequences such as fibrosis, cirrhosis, and cancer 8. Therefore, reducing cholesterol accumulation in the liver is a viable strategy for treating MASLD. One good example is that hyperosides can worsen MASLD by modulating cholesterol metabolism 9.

The ER is the primary organelle responsible for protein folding and post-translational modifications. Furthermore, it controls the cholesterol production and lipid-membrane biosynthesis as well as surviving and cell death signaling mechanisms in the cell 10. Cellular cholesterol homeostasis is greatly influenced by the cholesterol levels present in the ER, and excessive accumulation of cholesterol can cause ER stress 11. ER stress activation is a primary contributor to liver inflammation and fibrosis, which are hallmark indications of advanced fatty liver diseases, including liver cancer and MASH. Recently, pyroptosis has emerged as a novel form of programmed cell death 12. For pyroptosis, activation of the inflammasome is a prerequisite, and the NLRP3 inflammasome has been the subject of intensive scrutiny 13. NLRP3 is typically expressed in the ER under normal physiological conditions 14. Several studies have shown that ER stress can activate the NLRP3 inflammasome in MASLD 15, 16. However, the intrinsic pathways that cause chronic ER stress and activation of NLRP3 in fatty liver remain unknown.

Caveolin-1 (CAV1) is a key protein scaffold that binds to various signaling molecules in the caveolae of the cell membrane 17, performing crucial functions in numerous cellular processes, such as cell signaling, membrane transport 18, cholesterol homeostasis 19, and vesicle transport 20. CAV1 is involved in various diseases, including cancer, diabetes, nervous system diseases, Alzheimer's disease and cardiovascular diseases. Recently reported Caveolin-1 has been involved in cholesterol homeostasis, regulates the cholesterol content in caveolae by binding to cholesterol 21.

The CAV1 scaffold domain peptide (CSD), which comprises amino acids 82 to 101 of CAV1, enables the biological function of CAV1 22. CSD peptide plays a role in the development of tumor and fibrotic diseases, which suggests that specific domains of caveolin may contribute to many of these dynamic features involved in regulating cell physiology and morphology. 23, 24. Our previous research indicated that CAV1 could alleviate oxidative stress and inflammation in the vasculature of people with MASLD, which exacerbated by acetaminophen 25. Currently, we have reason to believe that CAV1 contributes to metabolic stress and inflammatory processes. However, the specific role of CAV1 in the advancement of MASLD remains uncertain.

In the present study, we established a MASLD mouse model in CAV1 knockout mice as well as the wild-type (WT) controls to investigated the role of CAV1 in MASLD. We identified some causal relationship between CAV1 deficiency and cholesterol accumulation, ER stress, and pyroptosis in MASLD progression. Treatment with CSD could mitigate the progression of MASLD with attenuates hepatic steatosis, inflammation, and liver injury, which offering a potential therapeutic approach for the condition.

## Materials and Methods

### Animals

Male C57BL/6J WT and male C57BL/6J global CAV1-knockout (CAV1-KO) mice (aged 8-9 weeks, weighing 18-20 g) were acquired from Model Organisms (Shanghai, China). The mice were reared in a controlled environment with a 12-h light-dark cycle (maintained at 20±2 °C). The Ethics Committee of Anhui Medical University approved the animal experimentation protocol (No. LLSC20231211). The mice were randomly allocated into five groups (n = 6 per group): 1) WT with normal chow diet (WT-NCD), 2) WT with high-fat diet (WT-HFD), 3) CAV1-KO with NCD (KO-NCD), 4) CAV1-KO with HFD (KO-HFD), and 5) KO-HFD with CSD treatment (KO-HFD+CSD) groups.

The mice were fed either NCD diet (TP26312; contained 10% fat, 20% protein, and 70% carbohydrates) or HFD diet (TP26300; contained 21.2% fat, 19.8% protein, 49.1% carbohydrates, and 0.2% cholesterol) (both from Trophic, Nantong, China) for 16 weeks. At week 12, KO-HFD+CSD mice were intraperitoneally injected with CSD (4 mg/kg) daily for 4 weeks. The CSD sequence used was DGIWKASFTTFTVTKYWFYR (Sangon Biotech, Shanghai, China).

### Human liver sample

The study followed the ethical guidelines of Anhui Medical University (project license number: 83230381) including obtaining informed consent from the patients and approval from the respective review committee. Sixteen liver samples were recruited from the First Affiliated Hospital of Anhui Medical University (Anhui, China) from March 7 to July19, 2023. Among them (all have pathological diagnosis), three male patients and one female patient showed normal histology, four male patients and four female patients with MASLD, and two male patients and two female with fibrosis. The liver tissues from the four normal liver and two male and two female patients with MASLD underwent western blot analysis, whereas every liver samples underwent immunohistochemistry (IHC) and immunofluorescence (IF) analysis. The specific information about the human samples can be found in Table S1.

### Biochemical analysis

Serum samples from WT or CAV1-KO mice were collected in each group for detection after animal sacrificed. Serum triglyceride (TG), total cholesterol (TC), low-density lipoprotein cholesterol (LDL-C), high-density lipoprotein cholesterol (HDL-C), aspartate aminotransferase (AST), alanine aminotransferase (ALT), lactate dehydrogenase (LDH), glutathione (GSH), and malondialdehyde (MDA) were quantified using reagent kits (Jiancheng, Nanjing, China).

### Measurement of cytokine levels

Serum interleukin IL-1β and IL-18 levels were quantified using an enzyme-linked immunosorbent assay (ELISA) kit (ColorfulGene, Wuhan, China), following the manufacturer's instructions.

### Histopathology and immunohistochemical staining

Paraffin and frozen tissue sections, with a thickness of 5 µm, were stained with hematoxylin and eosin (HE), Oil red O, and Masson staining.

Immunohistochemical staining was performed using paraffin sections with a thickness of 4 µm. Tissue slices were incubated with the primary antibody overnight, followed by incubation with the secondary antibody for 1 h. Subsequently, DAB and hematoxylin staining were performed, and the slides were examined using a slide scanner (3DHISTECH, Budapest, Hungary). All antibodies used are listed in Table S2.

### Immunofluorescence (IF)

Paraffin sections of 4 µm thickness were used for immunofluorescence detection. Initially, the sections were repaired and blocked, and were then subjected to incubation with the primary antibody overnight at 4 °C. Subsequently, they were incubated with fluorescent secondary antibody at room temperature for 2 h. The nuclei were then stained with 4',6-diamidino-2-phenylindole for 10 min. All images were captured using an inverted fluorescence microscope (Leica, Bensheim, Germany).

### Transmission electron microscopy (TEM)

Mouse liver tissue slices were fixed for 2 h at 4 °C with Gluta fixation solution, embedded in epoxy resin, cut to 50-70 µm using an ultramicrotome, and then observed using TEM (Hitachi, Tokyo, Japan).

### Filipin staining

After treatment, the cells were fixed with 4% paraformaldehyde at room temperature for 1 h. Subsequently, they were stained with 12.7 µM filipin (B6034; ApexBio, USA) for 1 h, washed three times with phosphate buffer solution, and then placed on a slide. Finally, the images were captured using a confocal laser microscope (Zeiss Microsystems, Oberkochen, Germany) with 405 nm excitation lasers.

### Cell culture

Alpha mouse liver 12 (AML-12) cells were obtained from the cell bank of the Chinese Academy of Sciences in Shanghai and maintained in Dulbecco's modified Eagle medium/F12 (Gibco, USA) supplemented with 10% fetal bovine serum (Bio-channel, Nanjing, China), 1% penicillin streptomycin solution, 10 µg/ml insulin, 5.5 µg/ml transferrin, 5 ng/ml selenium, and 40 ng/ml dexamethasone under the standard conditions of 37 °C and 5% CO_2_. To induce hepatocyte steatosis, we used a mixture of free fatty acids comprising palmitic acid and oleic acid in a 1:2 ratio (P/O), achieving a final concentration of 1 mM; the cells were then cultured for 24 h.

### Cell transfection and inhibitor administration

Small interfering RNA (SiRNA) of CAV1 and a negative control SiRNA were synthesized by GenePharma Co. Ltd. (Shanghai, China). The sequences were as follows: CAV1 SiRNA, sense 5'-CUGUGACCCACUCUUUGAATT-3' and antisense 5'-UUCAAAGAGUGGGUCACAGTT-3'. The GV146-CAV1 plasmid from GeneChem (Shanghai, China) was used to overexpress CAV1.

The CAV1 SiRNA reagent and plasmid mixture were then transfected into AML-12 cells with jetPRIME (Polyplus-jetPRIME, Illkirch, France) for a duration of 6 h. Cells were treated with mixture of free fatty acids (FAA). Methyl-β-cyclodextrin (MβCD, 4 mM/L), a cholesterol-depleting reagent (MCE, Shanghai, China), and GW4064 (1 μmol/L), an FXR (farnesoid X receptor, gene name NR1H4) agonist (Beyotime, Shanghai, China), were administered 1 h before FFA stimulation to investigate the involvement of cholesterol and FXR/NR1H4 in the protective effect of CAV1. The treated cells were then collected for subsequent experiments.

### Western blotting

The proteins from liver tissues and cells were extracted using RIPA lysis buffer, which contained 1% protease and phosphatase inhibitors (Solarbio, Beijing, China).

The sample was quantified for protein and separated using 8%-12.5% sodium dodecyl sulfate-polyacrylamide gel electrophoresis. Separated proteins were transferred to a polyvinylidene fluoride (PVDF) membrane (Millipore, Billerica, MA, USA), which then incubated with skimmed milk for 3 h, incubating with specific antibodies overnight at 4 °C thereafter.

The PVDF membrane was rinsed three times with tris-buffered saline with Tween 20 and then exposed to a secondary antibody (1:5000, ZS-GB, Beijing, China) at room temperature for 1 h. Subsequently, the protein bands were visualized using a chemiluminescence kit (ECL; NCM Biotech, Su Zhou, China) and quantified using ImageJ software. All antibodies used are listed in Table S2.

### RNA isolation and qRT-PCR

AML-12 total RNA was extracted using TRIzol reagent (Takara, Shiga, Japan). Subsequently, the RNA was converted to cDNA using a Takara kit (Takara). Furthermore, we performed reverse transcription and quantitative real-time PCR (qRT-PCR) using a commercially available kit (Takara). The quantification of mRNA levels was performed using the 2^-ΔΔCT^ method. For reference, the primer sequences are provided in Table S3.

### Statistical analysis

The experimental data were analyzed using GraphPad Prism Software version 9.0 (GraphPad Prism Software, CA, USA) and SPSS version 18.0 (SPSS Inc., Chicago, Illinois, USA). Data are presented as the mean ± standard deviation (SD). To compare multiple groups, we used a one-way analysis of variance (ANOVA) and set statistical significance at *P <* 0.05.

## Results

### CAV1 is downregulated in patients with MASLD and mouse models of MASLD

**Figure 1:**
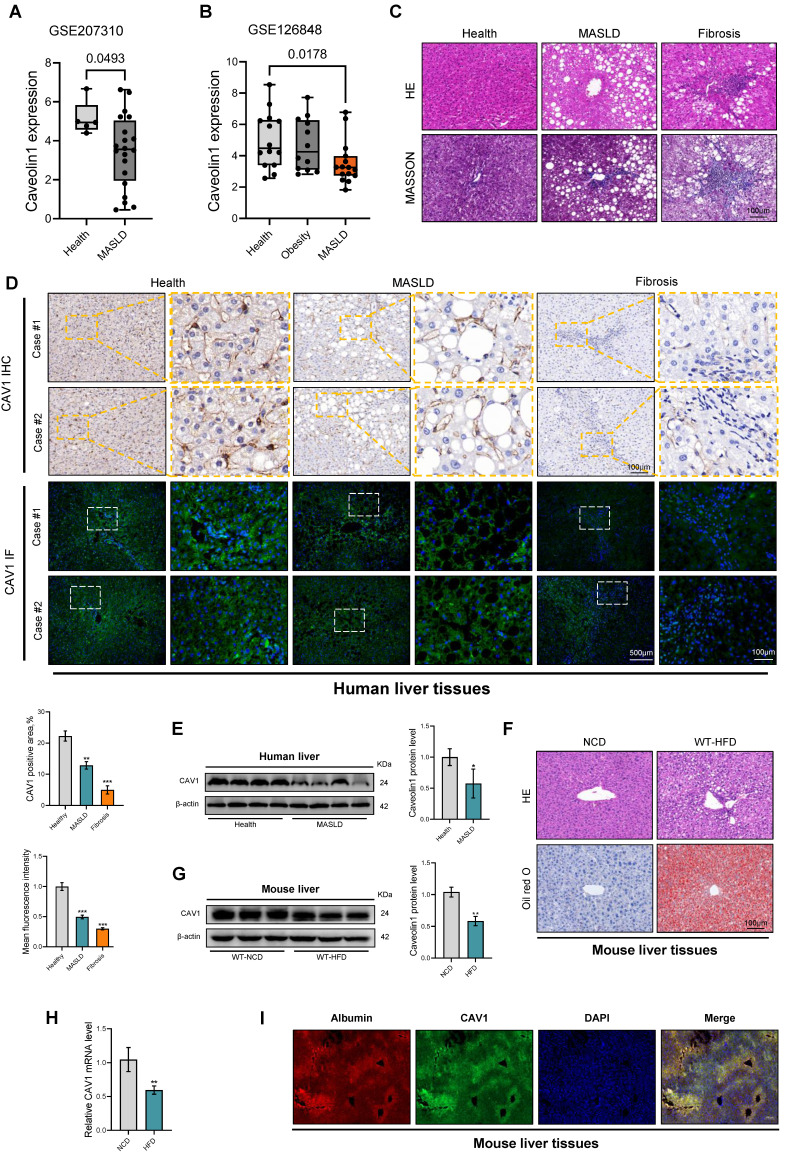
** CAV1 is downregulated in MASLD, Fibrosis patients and mouse models of MASLD.** (A) Relative CAV1 mRNA levels of health (n = 5), MASLD (n = 25) in GSE207310 datasets. (B) Relative CAV1 mRNA levels of health (n = 14), obesity (n = 12) and MASLD (n = 25) in GSE126848 datasets. (C) Representative H&E and MASSON staining of Human liver tissue sections. Scale bars, 100 µm. (D) Representative immunohistochemical and immunofluorescence staining and quantification of CAV1 in normal individuals and patients with MASLD and Fibrosis (n = 4). (E) Representative immunoblot and quantification of CAV1 protein in human livers (n = 4). Data are the mean±SD; ^*^P < 0.05, ^**^P < 0.01, ^***^P < 0.001 vs. Healthy. (F) Representative H&E and Oil red staining of WT-NCD and WT-HFD mouse liver tissue sections. Scale bars, 100 µm. (G) Representative immunoblot and quantification of CAV1 protein in mouse liver (n = 3). (H) CAV1 mRNA levels in mouse liver (n = 3). Data are the mean±SD; ^**^P < 0.01 vs. WT-NCD. (I) Albumin and CAV1 colocalized in the hepatocytes of liver by double immunofluorescence (Scale bar = 100 μm).

To investigate the role of CAV1 in the progression of MASLD, we assessed its expression in two cohorts of patients with MASLD obtained from the Gene Expression Omnibus Datasets (GSE207310 and GSE126848). Bioinformatics analysis confirmed that the mRNA level of CAV1 in the livers of patients with MASLD was lower than that in healthy individuals. (Fig. 1A, B). IHC and IF analyses of liver tissue sections revealed a large decrease of CAV1 protein expression in individuals with MASLD and fibrosis compared to healthy participants (Fig. 1C, D). Similarly, western blot analysis showed reduced expression of the CAV1 protein in the liver of individuals with MASLD (Fig. 1E). As expected, it was found that the mRNA and protein levels of CAV1 in the livers of mice decreased after 16 weeks of HFD feeding (Fig. 1 F-H). Notably, IF double staining showed that there was a typical colocalization of CAV1 and hepatocyte albumin immunoreactivity in liver tissues (Fig 1 I).

### Loss of CAV1 exacerbates HFD-induced liver injury and hepatic steatosis in mice and accelerated the progression of MASLD

**Figure 2:**
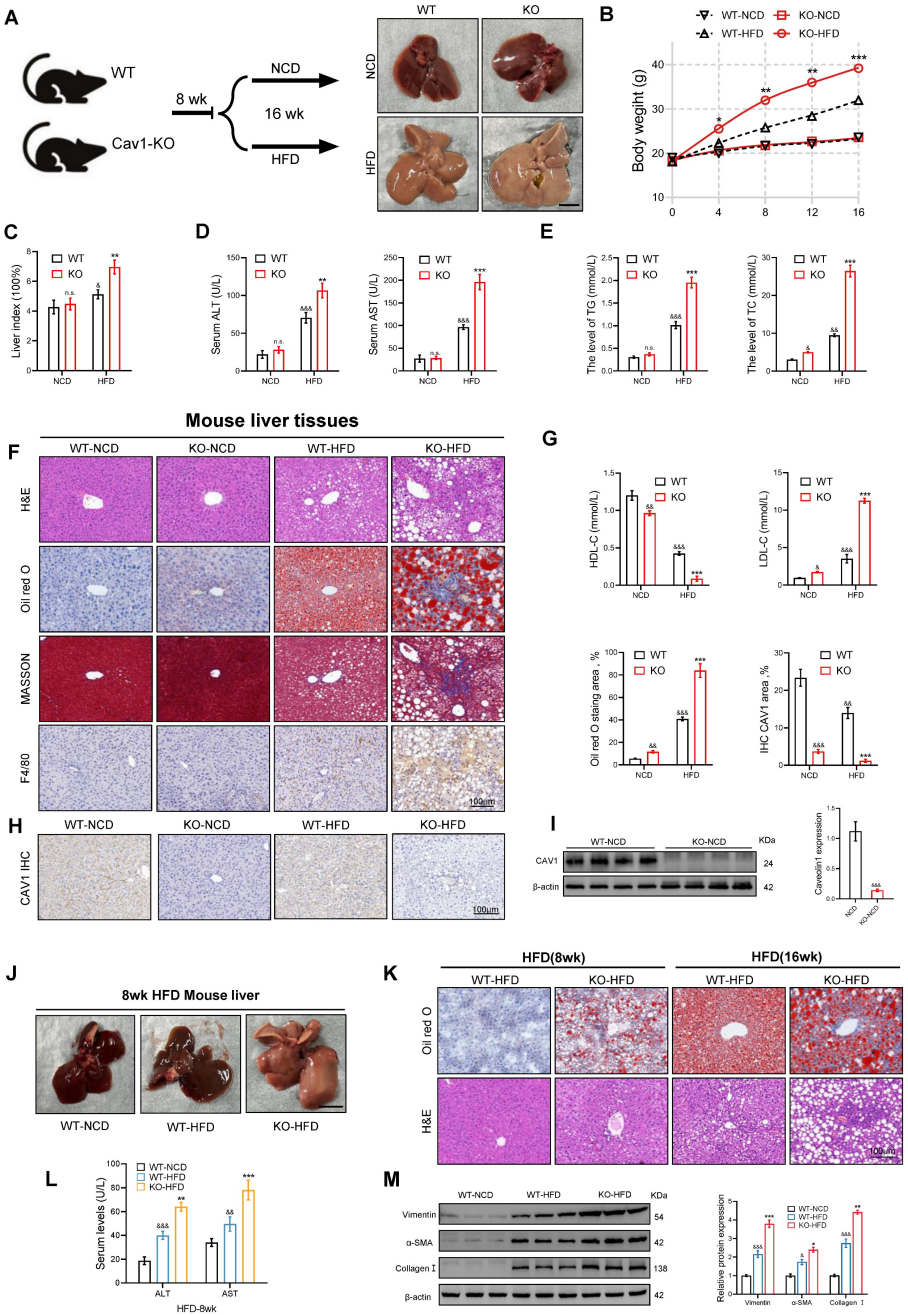
** Deletion of CAV1 aggravates HFD-induced liver injury and steatosis in MASLD mice.** (A) Animal experiment procedure. (B) Body weight was measured every 4 weeks from 0 to 16 weeks (n = 5). (C) Liver index of WT/KO-NCD and -HFD mice (n = 5). (D) Serum ALT and AST levels of WT/KO-NCD and -HFD mice (n = 5). (E) Serum TG and TC levels of WT/KO-NCD and -HFD mice (n = 5). (F) Representative H&E, Oil red and F4/80 immunohistochemical and MASSON staining and Oil red quantification of mouse liver tissue sections. Scale bars, 100 µm (n = 3). (G) Serum LDL-C and HDL-C levels of WT/KO-NCD and -HFD mice (n = 5). (H) Representative immunohistochemical staining and quantification of CAV1 in WT/KO-NCD and -HFD mouse livers (n = 5). (I) Representative immunoblot and quantification of CAV1 protein in WT-NCD and KO-NCD mouse livers (n = 4). (J) Representative liver morphological images. Scale bars, 1 cm. (K) Representative H&E and Oil red staining of mouse liver tissue sections. Scale bars, 100 µm. (L) Serum ALT and AST levels of WT-NCD, WT-HFD and KO-HFD mice (n = 5). (M) Representative immunoblot and quantification of Vimentin, α-SMA and Collagen I proteins in WT-NCD, WT-HFD and KO-HFD mouse livers (n = 5). Data are the mean±SD; ^&^P < 0.05, ^&&^P < 0.01, ^&&&^P < 0.001 vs. WT-NCD group; ^*^P < 0.05, ^**^P < 0.01, ^***^P < 0.001 vs. WT-HFD group. (J, K and L for 8 weeks, not specified, all for 16 weeks).

To investigate the role of CAV1 in MASLD, we fed eight-week-old wild-type (WT) and CAV1-KO mice NCD or HFD for 16 weeks (Fig. 2A). As the modeling time increased, the body weight of the CAV1-KO mice fed the HFD diet was higher than that of WT mice (Fig. 2B). CAV1-KO mice exhibited a higher consumption of high-calorie food within the same time frame, and appeared to display lethargic behavior. This may contribute to the accelerated body weight associated with CAV1 deficiency. The liver index serves as an indicator of both liver damage and fat accumulation within the organs. After 16 weeks of HFD feeding, the study revealed an increase in liver index in CAV1-KO mice fed HFD compared to WT HFD mice (Fig. 2C). CAV1 loss increased plasma levels of TG, TC, ALT, AST, and LDL-C and decreased HDL-C levels. In mice fed a NCD, CAV1 loss increased the plasma levels of TC and LDL-C and decreased the levels of HDL-C (Fig. 2D, E, G). Therefore, the lack of CAV1 could influence cholesterol homeostasis, even when on an NCD diet.

The livers of CAV1-KO mice showed more severe lipid deposition, inflammatory cell infiltration, and fibrosis, as demonstrated by Oil Red O, HE, F4/80 immunohistochemical and Masson staining (Fig. 2F). The correlation between the loss of CAV1 and the results was derived by immunohistochemical staining and western blotting (Fig. 2H, I). After 8 weeks of HFD feeding, CAV1-KO mice already exhibited mild fat deposition and inflammatory cell infiltration, along with elevated plasma ALT and AST levels (Fig. 2J-L). After 16 weeks of HFD feeding, CAV1-KO mice showed higher expression of fibrosis-related proteins in the liver than that of WT mice (Fig. 2M).

### The absence of CAV1 reduces the efflux of cholesterol and increases the accumulation of cholesterol in the liver

**Figure 3:**
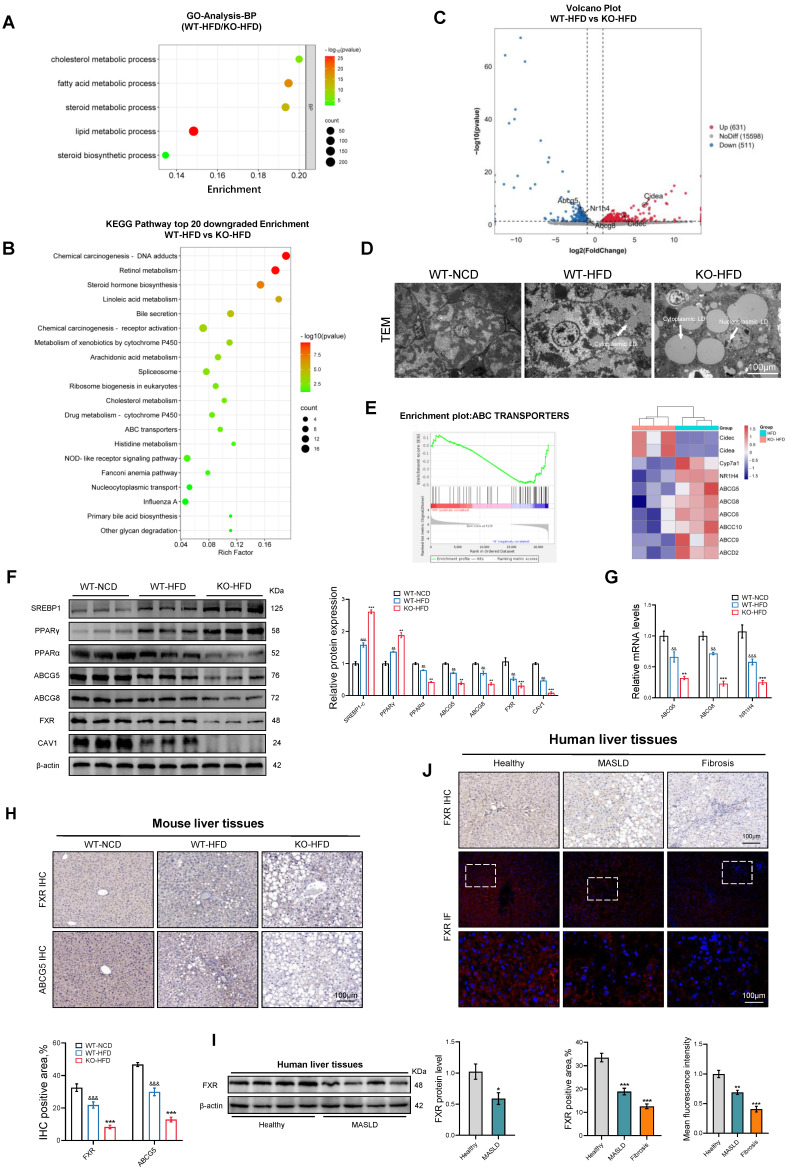
** Loss of CAV1 promotes cholesterol accumulation according to transcriptome analyses.** RNA sequencing was performed on the livers of WT (n = 3) and Cav1-KO (n = 5) mice fed the HFD. (A) Gene Ontology analysis of biological processes. (B) Volcano plot representation of significantly up- and downregulated genes. (C) KEGG enriched pathways (top 20 downregulated) of DEPS between WT-HFD and KO-HFD mice. (D) Representative TEM images of liver sections from WT-NCD, WT-HFD and KO-HFD mice. Scale bars, 100 μm. (E) Gene set enrichment analysis plot and Heatmap of relative genes. (F) Representative immunoblot and quantification of SREBP1, PPARγ, PPARα, ABCG5, ABCG8, FXR, CAV1 proteins in WT-NCD, WT-HFD and KO-HFD mouse livers (n = 3). (G) ABCG5, ABCG8 and NR1H4 mRNA levels in WT-NCD, WT-HFD and KO-HFD mouse livers (n = 3). (H) Representative immunohistochemical staining and quantification of FXR and ABCG5 in WT-NCD, WT-HFD and KO-HFD mouse livers (n = 3). Data are the mean±SD; ^&&^P < 0.01, ^&&&^P < 0.001 vs. WT-NCD group; ^**^P < 0.01, ^***^P < 0.001 vs. WT-HFD group. (I) Representative immunoblot and quantification of FXR protein in human livers (n = 4). (J) Representative immunohistochemical, immunofluorescence staining and quantification of FXR in normal individuals and patients with MASLD and Fibrosis (n = 3). Data are the mean±SD; ^*^P < 0.05, ^**^P < 0.01, ^***^P < 0.001 vs. Healthy.

To investigate the role of CAV1 in the progression of MASLD, we conducted RNA-seq analysis on liver samples from WT and CAV1-KO mice fed an HFD. Gene ontology (GO) analysis revealed an enrichment of lipid and cholesterol metabolism in CAV1-KO mice fed HFD (Fig. 3A). Furthermore, Kyoto Encyclopedia of Genes and Genomes (KEGG) analysis indicated a decrease in cholesterol metabolism in CAV1-KO mice fed an HFD (Fig. 3B). The volcano map indicates that the expression of the CIDEA and CIDEC genes, which are related to lipid drop synthesis in CAV1-KO mice, were upregulated. In contrast, the expression of NR1H4 and its downstream cholesterol transporters, ABCG5 and ABCG8, was downregulated (Fig. 3C). After obtaining this information, we are eager to verify our animal results. TEM revealed that cytoplasmic lipid droplets in HFD-fed CAV1-KO mice were larger and more numerous; lipid droplets were also present in the nucleus (Fig. 3D). Gene Set Enrichment Analysis (GSEA) revealed a downregulation of the ABC transporter signaling pathway in CAV1-KO mice (Fig. 3E). Consistent with these results, the protein expressions of SREBP1C and PPARγ increased in liver tissues of mice fed an HFD after CAV1 knockout, whereas the protein expressions of PPARα, ABCG5, ABCG8, and FXR (gene name NR1H4) decreased (Fig. 3F). Simultaneously, the mRNA levels of ABCG5, ABCG8, and NR1H4 decreased (Fig. 3G). The results of the IHC tests showed a decrease in the protein levels of FXR and ABCG5 in the liver after CAV1 knockout (Fig. 3H). Furthermore, the IHC and IF results also indicated a decrease in the expression of FXR in liver samples from patients with MASLD and fibrosis (Fig. 3I, J).

**Figure 4:**
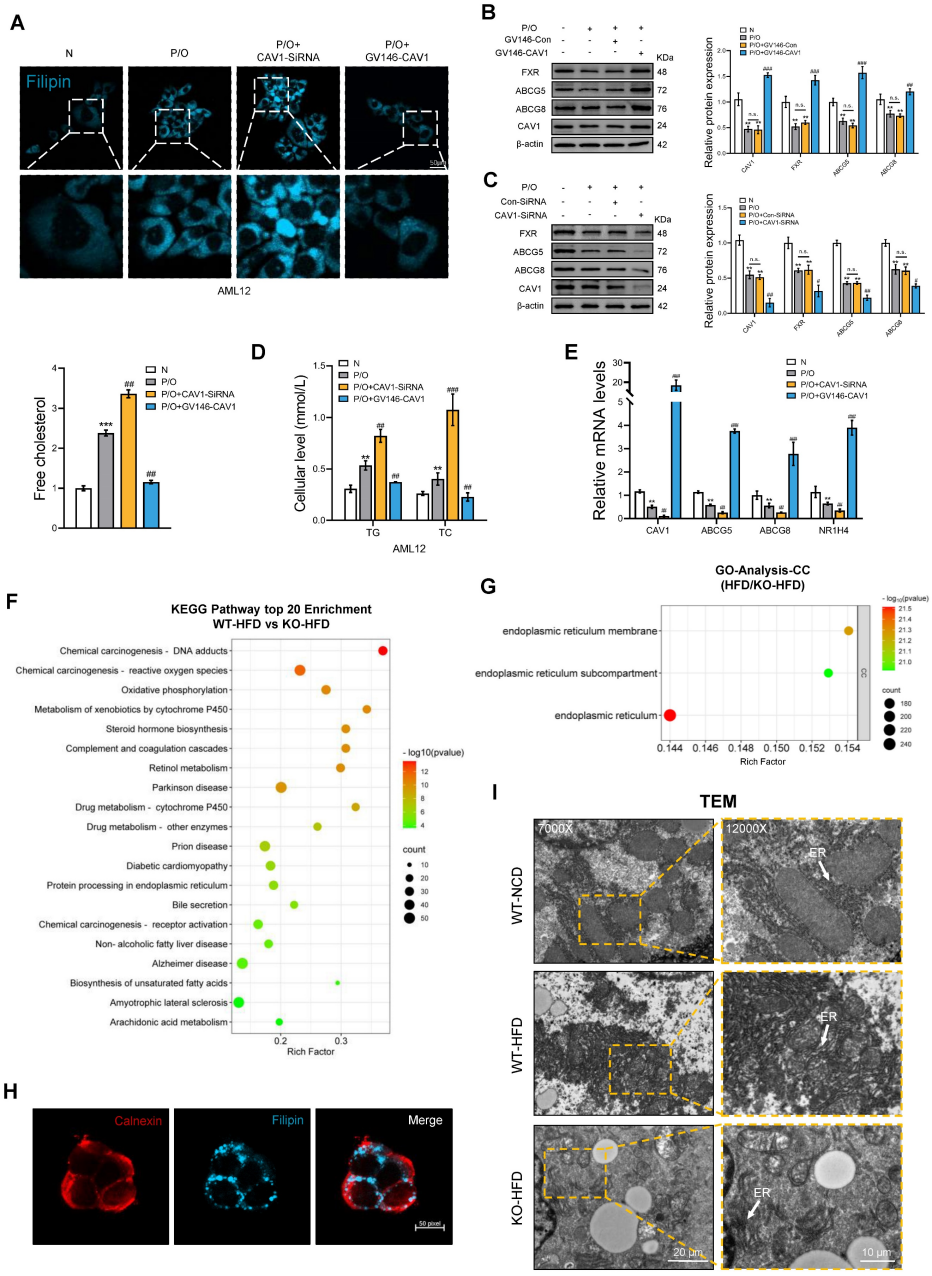
** The accumulation of cholesterol results in the dysfunction of the endoplasmic reticulum.** (A) Cellular Free cholesterol content was determined using filipin staining analyzed by confocal microscopy and quantification. Scale bar, 50 μm (n = 3). (B) Representative immunoblot and quantification of FXR, ABCG5, and ABCG8 proteins in P/O stimulated AML-12 hepatocytes treated with GV146-CAV1 (n = 3). (C) Representative immunoblot and quantification of FXR, ABCG5, and ABCG8 proteins in P/O stimulated AML-12 hepatocytes treated with CAV1-SiRNA (n = 3). (D) Intracellular TG and TC levels in AML12 cells (n = 3). (E) Relative mRNA expression of CAV1, ABCG5, ABCG8 and NR1H4 was determined from AML-12 hepatocytes treated with P/O, GV146-CAV1 and CAV1-SiRNA (n = 3). (F) KEGG enriched pathways (top 20 upregulated) of DEPS between WT-HFD and KO-HFD mice. (G) Gene Ontology analysis of all significantly changed genes in cell component. (H) Representative immunofluorescence staining of Calnexin and Filipin in P/O stimulated AML-12 hepatocytes treated with CAV1-SiRNA. Scale bar, 50 pixel. (I) Representative TEM images of liver sections from WT-NCD, WT-HFD and KO-HFD mice. Scale bars, 10 μm. White arrows indicated the endoplasmic reticulum of cells. Data are the mean±SD. ns, no significance; ^*^P < 0.05, ^**^P < 0.01, ^***^P < 0.001 vs. Normal group; ^#^P < 0.05, ^##^P < 0.01, ^###^P < 0.001 vs. P/O group.

*In vitro*, after silencing the CAV1 gene in AML12 cells, we observed an increased accumulation of FC, TC and TG in the intracellular, which decreased after the overexpression of CAV1 (Fig. 4A, D). Furthermore, the protein and mRNA levels of NR1H4 and its downstream cholesterol transporter ABCG5/ABCG8 decreased and increased, respectively, following CAV1 silencing and overexpression (Fig. 4B, C, E).

### CAV1 deficiency promotes ER stress-induced unfolded protein response (UPR)

We investigated the effect of CAV1 deficiency on cholesterol accumulation. KEGG analysis showed that HFD-fed CAV1-KO mice had a higher enrichment of endoplasmic reticulum protein synthesis and cytochrome P450 enzyme drug metabolism compared to those in their WT counterparts (Fig. 4F). GO-CC analysis also revealed a decrease in the ER, ER subcompartments, and ER membranes in CAV1-KO mice (Fig. 4G). Laser confocal microscopy showed the co-localization of the ER marker calnexin with cholesterol, providing evidence that the ER stores cholesterol intracellularly (Fig. 4H). TEM provided a more direct visualization. In mice fed an HFD, the ER in WT mice displayed swelling; the ER in CAV1-KO mice showed even more pronounced swelling and extensive dissolution (Fig. 4I). These results suggest that the absence of CAV1 worsens the accumulation of cholesterol in the ER and perturbs hepatocyte ER homeostasis.

**Figure 5:**
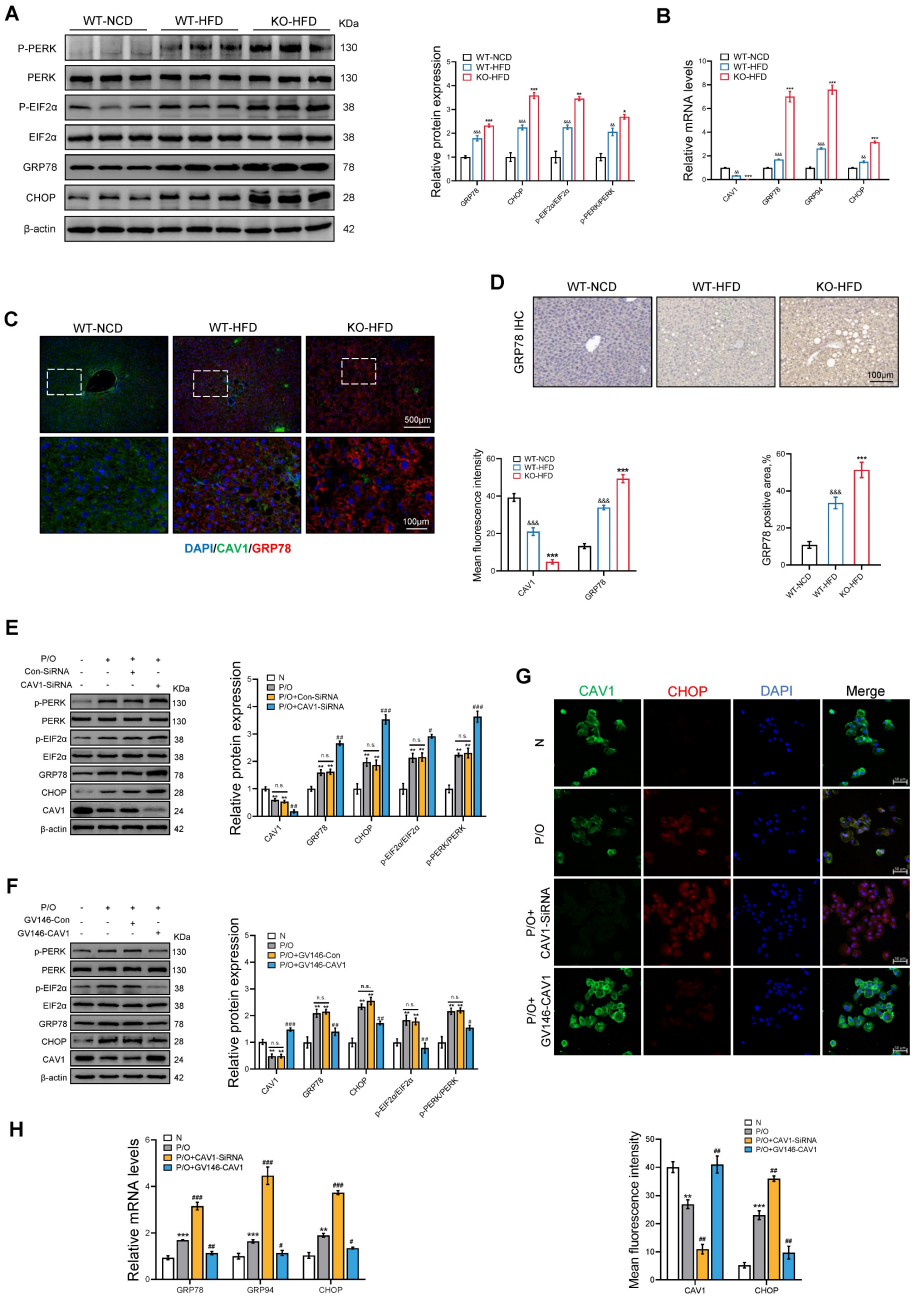
** CAV1 deficiency results in the accumulation of cholesterol, leading to more severe ER stress.** (A) Representative immunoblot and quantification of ER Stress relative proteins in WT-NCD, WT-HFD and KO-HFD mouse livers (n = 3). (B) CAV1, GRP78, GRP94, XBP1 and CHOP mRNA levels in WT-NCD, WT-HFD and KO-HFD mouse livers (n = 3). (C) Representative immunofluorescence staining and mean fluorescence intensity of GRP78 and CAV1 in WT-NCD, WT-HFD and KO-HFD mouse livers (n = 3). (D) Representative immunohistochemical staining and quantification of GRP78 in WT-NCD, WT-HFD and KO-HFD mouse livers (n = 3). Data are the mean±SD; ^&&^P < 0.01, ^&&&^P < 0.001 vs. WT-NCD group; ^**^P < 0.01, ^***^P < 0.001 vs. WT-HFD group. (E) Representative immunoblot and quantification of ER Stress relative proteins in P/O stimulated AML-12 hepatocytes treated with CAV1-SiRNA (n = 3). (F) Representative immunoblot and quantification of ER Stress relative proteins in P/O stimulated AML-12 hepatocytes treated with GV146-CAV1 (n = 3). (G) Representative immunofluorescence staining and mean fluorescence intensity of CHOP and CAV1 in P/O stimulated AML-12 hepatocytes treated with GV146-CAV1 and CAV1-SiRNA. Scale bar, 50 μm (n = 3). (H) Relative mRNA expression of GRP78, GRP94 and CHOP was determined from AML-12 hepatocytes treated with P/O, GV146-CAV1 and CAV1-SiRNA (n = 3). Data are the mean±SD; ns, no significance; ^*^P < 0.05, ^**^P < 0.01, ^***^P < 0.001 vs. Normal group; ^#^P < 0.05, ^##^P < 0.01, ^###^P < 0.001 vs. P/O group.

Furthermore, we investigated the markers associated with ER stress in mice fed an HFD, and found that the protein and mRNA levels of ER stress-related indicators such as GRP78 and CHOP were elevated in CAV1-KO mice compared to those in WT mice (Fig. 5A, B). The immunohistochemical (IHC) and immunofluorescence (IF) results for GRP78 also showed more severe ER stress in Cav1-KO mice (Fig. 5C, D). *In vitro* experiments showed a notable increase in protein and mRNA expression of ER stress-related indicators when CAV1 was silenced (Fig. 5E, G, H).

In contrast, overexpression of CAV1 through plasmid transfection resulted in decreased protein and mRNA levels of ER stress-related indicators (Fig. 5F, G, H). These findings suggest that CAV1 deficiency leads to cholesterol accumulation and severe ER stress.

### The deficiency of CAV1 promotes the activation of the NLRP3 inflammasome

**Figure 6:**
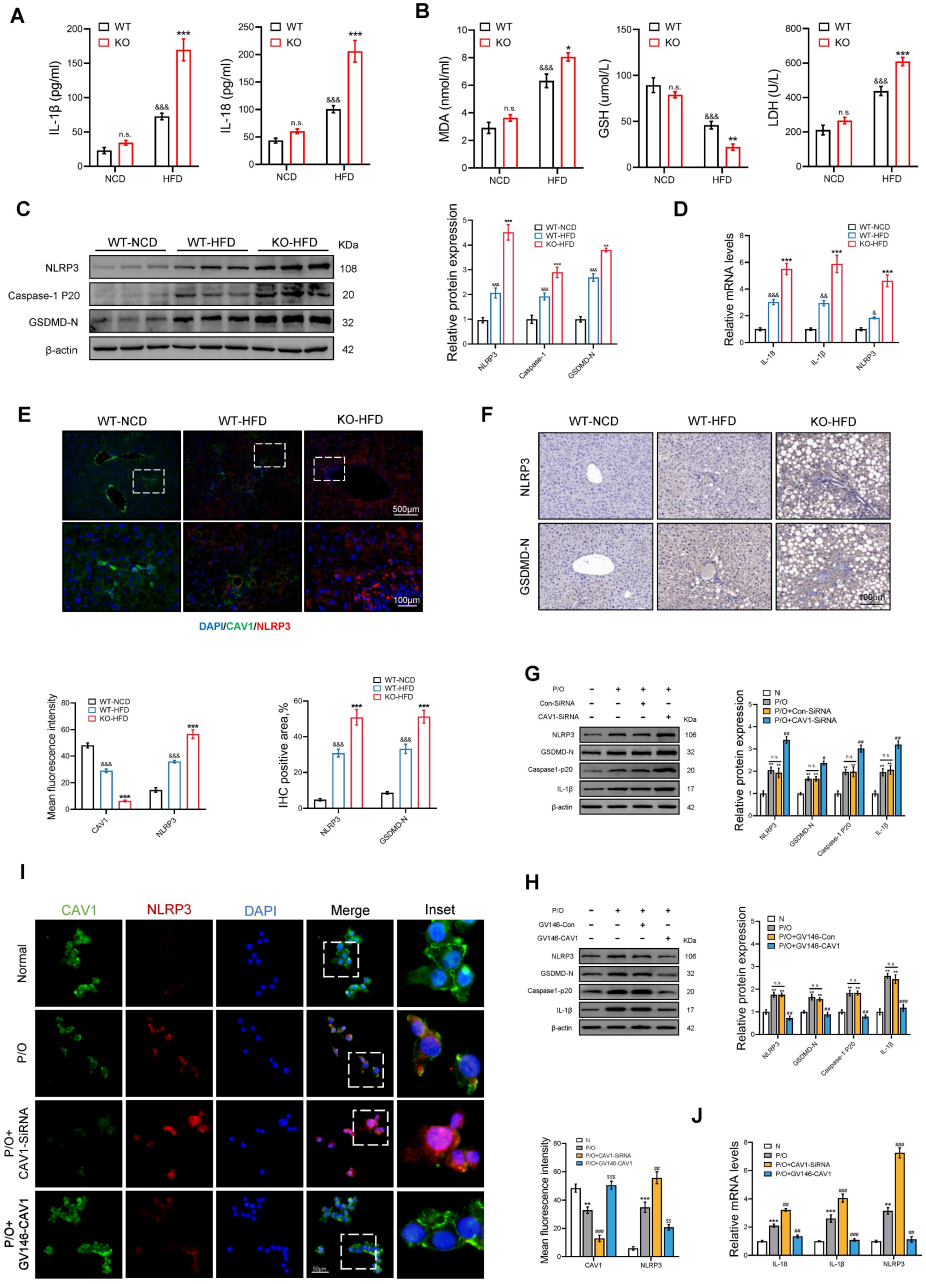
** CAV1 deficiency promotes NLRP3 inflammasome activation and pyroptosis.** (A) ELISA measurement of serum IL-1β and IL-18 (n = 5). (B) Serum MDA, GSH and LDH levels of WT/KO-NCD and -HFD mice (n = 5). (C) Representative immunoblot and quantification of NLRP3, Caspase-1 p20, GSDMD-N proteins in WT-NCD, WT-HFD and KO-HFD mouse livers (n = 3). (D) IL-1β, IL-18 and NLRP3 mRNA levels in WT-NCD, WT-HFD and KO-HFD mouse livers (n = 3). (E) Representative immunofluorescence staining and mean fluorescence intensity of NLRP3/CAV1 in WT-NCD, WT-HFD and KO-HFD mouse livers (n = 3). (F) Representative immunohistochemical staining and quantification of NLRP3 and GSDMD-N in WT-NCD, WT-HFD and KO-HFD mouse livers (n = 3). Data are the mean±SD; ^&^P < 0.05, ^&&^P < 0.01, ^&&&^P < 0.001 vs. WT-NCD group; ^*^P < 0.05, ^**^P < 0.01, ^***^P < 0.001 vs. WT-HFD group. (G) Representative immunoblot and quantification of NLRP3, GSDMD-N, Caspase-1 p20, and IL-1β proteins in P/O stimulated AML-12 hepatocytes treated with CAV1-SiRNA (n = 3). (H) Representative immunoblot and quantification of NLRP3, GSDMD-N, Caspase-1 p20, and IL-1β proteins in P/O stimulated AML-12 hepatocytes treated with GV146-CAV1 (n = 3). (I) Representative immunofluorescence staining and mean fluorescence intensity of NLRP3/CAV1 in P/O stimulated AML-12 hepatocytes treated with GV146-CAV1 and CAV1-SiRNA. Scale bar, 50 μm (n = 3). (J) Relative mRNA expression of IL-18, IL-1β and NLRP3 was determined from AML-12 hepatocytes treated with P/O, GV146-CAV1 and CAV1-SiRNA (n = 3). Data are the mean±SD; ^*^P < 0.05,^ **^P < 0.01, ^***^P < 0.001 vs. Normal group; ^#^P < 0.05, ^##^P < 0.01, ^###^P < 0.001 vs. P/O group.

We evaluated markers of inflammation and pyroptosis in mice. The ELISA results showed an increase in serum levels of IL-1β and IL-18 in CAV1-KO mice fed an HFD (Fig. 6A). Biochemical tests showed that CAV1-KO mice had higher serum levels of MDA and LDH and lower serum levels of GSH compared to WT mice (Fig. 6B). Moreover, CAV1-KO mice fed an HFD exhibited increased levels of protein and mRNA expression of NLRP3, Caspase-1 p20, GSDMD, which closely associated with liver pyroptosis (Fig. 6C, D). Furthermore, the results of IF and IHC demonstrated that deletion of CAV1 improved activation of the NLRP3 inflammasome (Fig. 6E, F).

The results obtained *in vivo* were consistent with those obtained from hepatocyte steatosis in AML-12 cells induced by P/O. An increase in the expression of NLRP3 and pyroptosis-related proteins and a simultaneous inhibition of CAV1 protein expression was observed in P/O-treated cells compared to the normal cell group. Furthermore, the intervention of CAV1 SiRNA resulted in a further reduction in CAV1 expression and an increase in the levels of NLRP3, pyroptosis-related proteins, and mRNA (Fig. 6G, J). In contrast, the plasmid intervention reduced NLRP3-mediated pyroptosis induced by P/O (Fig. 6H, J). Moreover, the IF staining results suggest that CAV1 regulates the activation of the NLRP3 inflammasome (Fig. 6I).

### Administration of CSD mitigates the liver injury resulting from CAV1 deficiency

**Figure 7:**
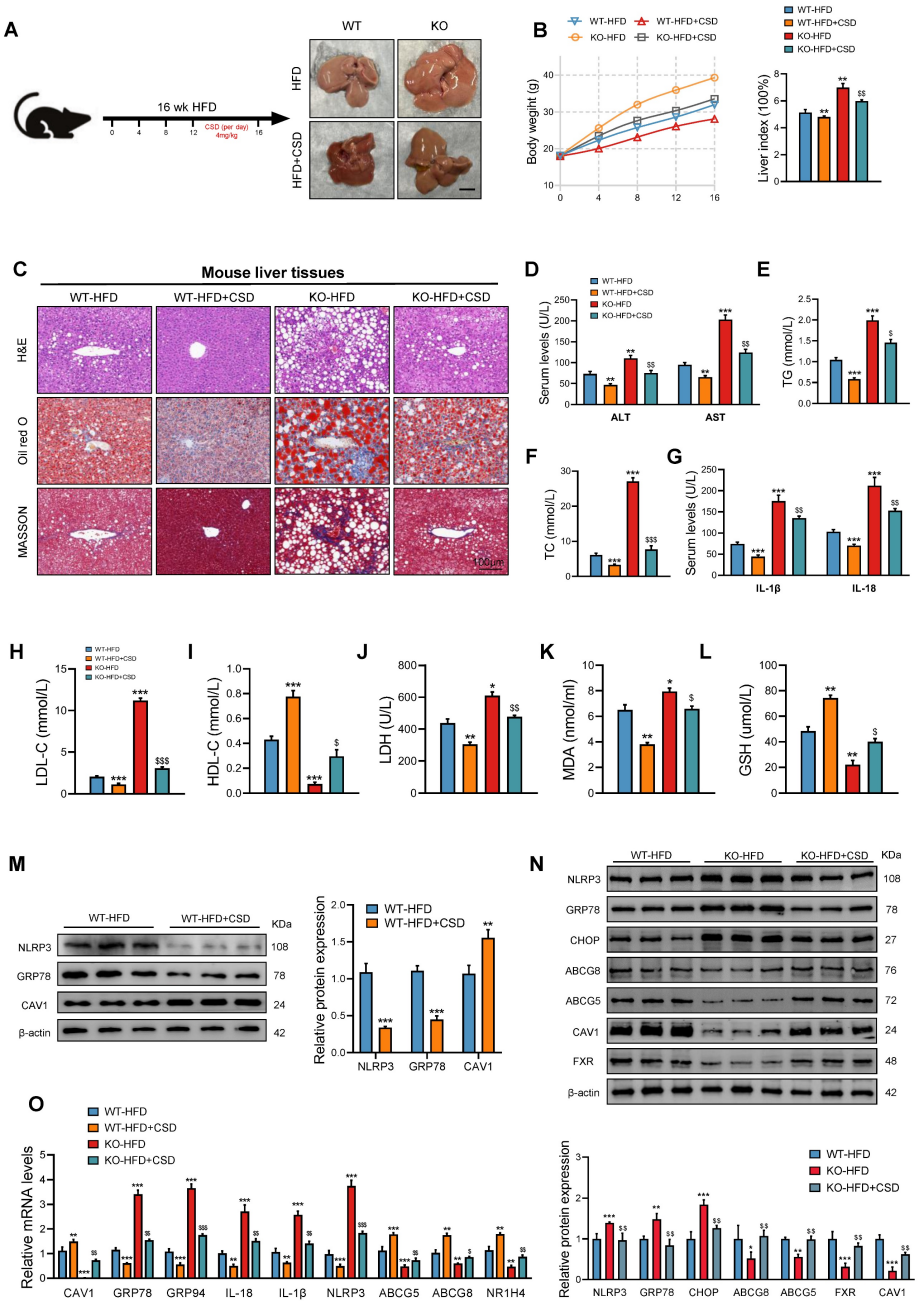
** The administration of CSD mitigates the liver injury resulting from CAV1 deficiency.** (A) Animal experiment procedure. (B) Body weight was measured every 4 weeks from 0 to 16 weeks and liver index of WT/KO-HFD and +CSD mice (n = 5). (C) Representative H&E, Oil red and MASSON staining of mouse liver tissue sections. Scale bars, 100 µm (n = 3). (D) Serum ALT and AST levels of WT/KO-HFD and CSD mice (n = 5). (E-F) Serum TG and TC levels of WT-HFD, KO-HFD and KO-HFD+CSD mice (n = 5). (G) ELISA measurement of serum IL-1β and IL-18 (n = 5). (H-I) Serum LDL-C and HDL-C levels of WT/KO-HFD and CSD mice (n = 5). (J) Serum LDH levels of WT/KO-HFD and CSD mice (n = 5). (K) Serum MAD levels of WT/KO-HFD and CSD mice (n = 5). (L) Serum GSH levels of WT/KO-HFD and CSD mice (n = 5). (M) Representative immunoblot and quantification of NLRP3, GRP78, and CAV1 proteins in WT-HFD and WT-HFD+CSD mouse livers (n = 3). (N) Representative immunoblot and quantification of NLRP3, GRP78, CHOP, ABCG8, ABCG5, CAV1 and FXR proteins in WT-HFD, KO-HFD and KO-HFD+CSD mouse livers (n = 3). (O) CAV1, GRP78, GRP94, IL-18, IL-1β, NLRP3, ABCG5, ABCG8 and NR1H4 mRNA levels in WT/KO-HFD and CSD mouse livers (n = 3). Data are the mean±SD. ^*^P < 0.05, ^**^P < 0.01, ^***^P < 0.001 vs. WT-HFD group; ^$^P < 0.05, ^$$^P < 0.01, ^$$$^P < 0.001 vs. KO-HFD group.

To further investigate CAV1's involvement in the progression of MASLD, we administered CSD (4.0 mg/kg body weight) chronically to HFD-fed mice daily for 4 weeks (Fig. 7A). At the end of the treatment period, the CSD group showed a marked decrease in body weight and liver index (Fig. 7B). Oil Red O and HE staining revealed that CAV1 deficiency exacerbated fat deposition, inflammatory cell infiltration, and fibrosis in the livers of mice, which were subsequently reversed by CSD treatment (Fig. 7C). The biochemical detection indices and ELISA results indicated that CSD reduced the levels of TG, TC, ALT, AST, IL-1β, IL-18, LDL-C, LDH, and MDA, while increasing the levels of HDL-C and GSH (Fig. 7D-L). Protein and mRNA levels of ER stress and pyroptosis-related indices decreased after CSD treatment. In contrast, the protein and mRNA levels of FXR/NR1H4 and its downstream mediator, cholesterol efflux ABCG5/ABCG8, increased after CSD treatment (Fig. 7M-O).

Treated with CSD in the absence of CAV1 ameliorated liver injury in CAV1-KO mice. Furthermore, the application of CSD could also delay the progression of MASLD in CAV1-KO mice.

### MβCD and FXR agonists GW4064 alleviate endoplasmic reticulum stress and pyroptosis *in vitro*

**Figure 8:**
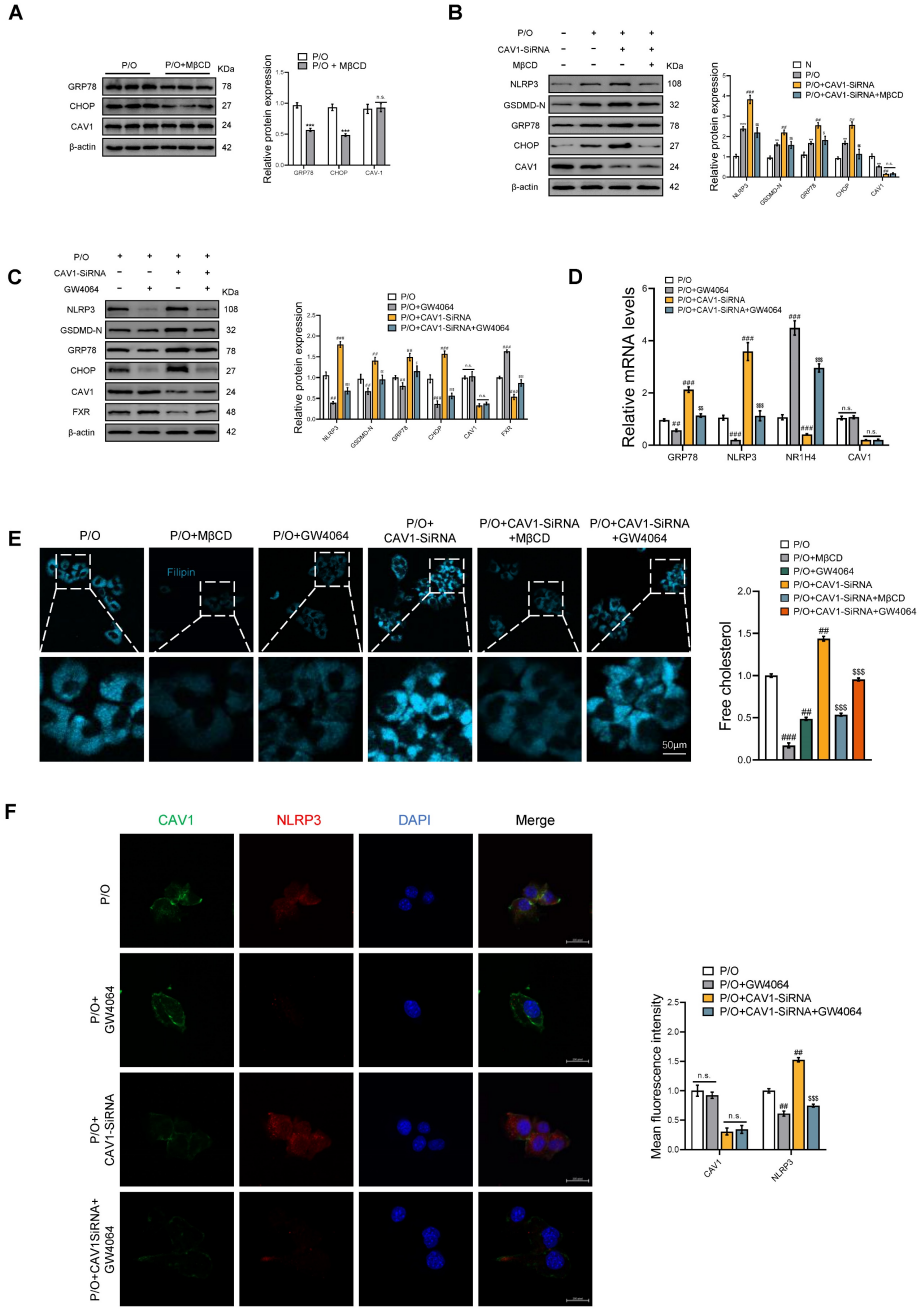
** The FXR agonist GW4064 alleviates ER stress and pyroptosis in P/O stimulated AML-12 hepatocytes treated with CAV1-SiRNA.** (A) Representative immunoblot and quantification of GRP78, CHOP and CAV1 proteins in P/O stimulated AML-12 hepatocytes treated with MβCD (n = 3). (B) Representative immunoblot and quantification of NLRP3, GSDMD-N, GRP78, CHOP and CAV1 proteins in P/O stimulated AML-12 hepatocytes treated with CAV1-SiRNA and MβCD (n = 3). (C) Representative immunoblot and quantification of NLRP3, GSDMD-N, GRP78, CHOP, CAV1 and FXR proteins in P/O stimulated AML-12 hepatocytes treated with CAV1-SiRNA and GW4064 (n = 3). (D) Relative mRNA expression of GRP78, NLRP3, NR1H4 and CAV1 was determined from AML-12 hepatocytes treated with P/O, GW4064 and CAV1-SiRNA (n = 3). (E) Cellular Free cholesterol content was determined using filipin staining analyzed by confocal microscopy and quantification. Scale bar, 50 μm (n = 3). (F) Representative immunofluorescence staining and mean fluorescence intensity of NLRP3/CAV1 in P/O stimulated AML-12 hepatocytes treated with GW4064 and CAV1-SiRNA. Scale bar, 200 pixels (n = 3). Data are the mean±SD. ns, no significance; ^**^P < 0.01, ^***^P < 0.001 vs. N group; ^##^P < 0.01, ^###^P < 0.001 vs. P/O group; ^$^P < 0.05, ^$$^P < 0.01, ^$$$^P < 0.001 vs. P/O+CAV1-SiRNA group.

*In vitro*, we have provided additional evidence that CAV1 regulates the disease process by controlling cholesterol efflux. First, we used MβCD, a cholesterol-depleting reagent, compared with the P/O group, although there was no significant change in the protein expression of CAV1, the protein levels of ER stress were reduced (Fig. 8A). Compared with the P/O+CAV1-SiRNA group, there was also no significant change in the protein expression of CAV1 in P/O+CAV1-SiRNA+MβCD group, but the protein levels of ER stress and the related cell pyroptosis were reduced (Fig. 8B). Subsequently, we treated the P/O+CAV1-SiRNA group with GW4064, an agonist of FXR, resulting in comparable reductions in the protein and mRNA levels of ER stress and pyroptosis with no significant change in CAV1 expression (Fig. 8C, D). Filipin staining revealed a reduction in cellular cholesterol content in both the P/O+CAV1-SiRNA+MβCD and P/O+CAV1-SiRNA+GW4064 groups (Fig. 8E). Furthermore, the IF results demonstrated a notable decrease in the expression of the NLRP3 inflammasome in the GW4064 treated group (Fig. 8F).

## Discussion

Our study demonstrates that CAV1 plays a crucial role in the progression of MASLD. We observed a reduction in the expression of CAV1 in the livers of both mice and patients with MASLD. Furthermore, the deficiency of CAV1 in mouse models fed an HFD notably exacerbated hepatic steatosis, inflammation, and fibrosis. Mechanistic investigations revealed that loss of CAV1 leads to cholesterol accumulation, which subsequently induces ER stress and pyroptosis. These findings provide evidence that CAV1 depletion promotes the progression of MASLD.

Lipotoxicity is a key factor in the development of diseases, such as MASLD 26. As the liver progresses from a healthy state to MASLD and eventually to NASH, the levels of saturated fatty acids and cholesterol increase concurrently 27. CAV1 is a membrane protein that plays a role in various cellular processes. The activation of CAV1 inhibits lipid accumulation in hepatocytes, making it a promising target for investigating metabolic disorders 28. However, the precise role of CAV1 in the pathological progression of liver from MASLD to MASH remains unknown.

Cholesterol accumulation is an early event in the development of MASLD. Mounting evidence indicates a correlation between alterations in cholesterol homeostasis and the onset of MASLD. In individuals with MASLD, MASH and fibrosis progress concomitantly with hepatic cholesterol accumulation. Cholesterol-mediated liver inflammation is a critical step in the pathogenesis of MASLD, potentially promoting liver damage and eventually liver fibrosis, cirrhosis, and liver cancer. Inducing hepatic cholesterol accumulation in MASH worsens steatohepatitis and fibrosis, while correcting hepatic cholesterol overload ameliorates liver disease severity 29. Recent evidence suggests that cholesterol buildup promotes the progression of MASLD and plays a crucial role in the transition from MASLD to MASH 30. CAV1 regulates hepatic cholesterol distribution primarily through the regulation of cholesterol efflux 31. Our gain-of-function and loss-of-function experiments revealed that CAV1 deficiency downregulated cholesterol metabolism in MASLD, promoted cholesterol accumulation. FXR plays a crucial molecular role in maintaining hepatic homeostasis, and when activated, inhibits fatty acid synthesis, improves mitochondrial β-oxidation, and promotes cholesterol excretion 32. In atherosclerosis, the downregulation of FXR results in reduced expression of cholesterol transport proteins (ABCG5/ABCG8), ultimately affecting cholesterol efflux and causing cholesterol accumulation 33. The present study demonstrates that the loss of CAV1 reduces the expression of FXR/NR1H4, resulting in the accumulation of cholesterol in the liver, which triggering endoplasmic reticulum (ER) stress.

The ER is responsible for ensuring proper protein folding 34. Excessive cholesterol accumulation within the ER depletes calcium stores, leading to ER stress and subsequent activation of the UPR pathway 35. UPR activation is effective in repairing mild and transient forms of ER stress 36. However, if ER stress is severe and left unaddressed, it can lead to sustained activation of the UPR signaling pathway, ultimately resulting in inflammasome activation, cell death, and accelerated organ damage 37. Nevertheless, the role of CAV1 in the regulation of ER stress in various diseases remains a matter of debate. Díaz MI 38 and Zeng W 39 have conflicting views on the importance of CAV1 in ER stress. Current study presents novel evidence that CAV1 has been involved in the progression of MASLD induced by ER stress. Our findings suggest that the loss of CAV1 in both hepatocytes and liver tissues worsens ER injury and promotes ER stress due to cholesterol accumulation.

Pyroptosis is a form of pro-inflammatory programmed cell death that has been implicated in various diseases. Studies have identified the NLRP3 inflammasome as a key mediator of pyroptosis 40, and ER stress has also been observed to induce pyroptosis 41. Mechanistic investigations have revealed that cholesterol trafficking to the ER is necessary to activate the NLRP3 inflammasome 42. Recent studies have demonstrated the critical role of pyroptosis in diet-induced MASLD 43, 44. Our previous studies have demonstrated that CAV1 can improve acetaminophen-induced liver injury in MASLD by inhibiting pyroptosis 45. In this study, the absence of CAV1 in hepatocytes and liver tissues promotes the activation of the NLRP3 inflammasome.

Mechanistically, the lack of CAV1 may contribute to increased intracellular cholesterol accumulation, which induces ER stress and activates the NLRP3 inflammasome.

CSD, a stable analog of the active portion of CAV1, supplementing CSD can restore the expression of CAV1, function synergistically with cholesterol sequestration, a process also dependent on the CSD, to effect CAV1 signaling function through both lipid and protein interaction 46-47. Recent studies indicate that CSD accumulates in the ER, which mitigates ER stress during pulmonary fibrosis 48. However, whether CSD could alleviate ER stress in MASLD and its underlying mechanisms remains unclear. In our study, CAV1 function was supplemented by intraperitoneally injecting CSD, which effectively reversed cholesterol accumulation, ER stress, and pyroptosis exacerbated by CAV1 deficiency; thus, slowing the progression of MASLD.

The present study shows that MβCD, a widely used reagent to deplete cholesterol 49, reversed alterations in ER stress and pyroptosis levels mediated by CAV1 SiRNA, suggesting that cholesterol accumulation contributes to the regulatory effect of CAV1 on ER stress and pyroptosis. Furthermore, GW4064 (an FXR agonist) reduces cholesterol levels induced by an HFD 50, and increases cholesterol efflux by inducing the expression of FXR/NR1H4 and its downstream cholesterol transporter ABCG5/ABCG8 51. As expected, we observed that GW4064 treatment alleviated the exacerbation of ER stress and pyroptosis induced by CAV1 SiRNA, these results suggesting that FXR/NR1H4 serves as a crucial link in the regulation of Cav1-mediated ER stress and pyroptosis triggered by cholesterol accumulation.

In conclusion, the evidence presented in our study has identified CAV1 as a crucial regulator of cholesterol homeostasis in MASLD, who plays an important role in disease progression. However, further investigation is required to elucidate the specific underlying molecular mechanisms. Global CAV1 deficiency promotes hepatic cholesterol deposition in a murine model of MASLD, exacerbating ER stress and its mediated pyroptosis, thus accelerating MASLD progression. The hope is that supplementing CSD could reverse this change. Therefore, CAV1 is a promising therapeutic target for MASLD.

## Supplementary Material

Supplementary tables.

## References

[1] Ferguson D, Finck BN (2021). Emerging therapeutic approaches for the treatment of NAFLD and type 2 diabetes mellitus. Nat Rev Endocrinol.

[2] Younossi ZM, Golabi P, Paik JM, Henry A, Van Dongen C, Henry L (2023). The global epidemiology of nonalcoholic fatty liver disease (NAFLD) and nonalcoholic steatohepatitis (NASH): a systematic review. Hepatology.

[3] Sheka AC, Adeyi O, Thompson J, Hameed B, Crawford PA, Ikramuddin S (2020). Nonalcoholic Steatohepatitis: A Review. JAMA.

[4] Zhang T, Li H, Wang K, Xu B, Chen ZN, Bian H (2020). Deficiency of CD147 Attenuated Non-alcoholic Steatohepatitis Progression in an NLRP3-Dependent Manner. Front Cell Dev Biol.

[5] Itoh M, Tamura A, Kanai S, Tanaka M, Kanamori Y, Shirakawa I (2023). Lysosomal cholesterol overload in macrophages promotes liver fibrosis in a mouse model of NASH. J Exp Med.

[6] Dong J, Viswanathan S, Adami E, Singh BK, Chothani SP, Ng B (2021). Hepatocyte-specific IL11 cis-signaling drives lipotoxicity and underlies the transition from NAFLD to NASH. Nat Commun.

[7] Li H, Yu XH, Ou X, Ouyang XP, Tang CK (2021). Hepatic cholesterol transport and its role in non-alcoholic fatty liver disease and atherosclerosis. Prog Lipid Res.

[8] Ioannou GN (2016). The Role of Cholesterol in the Pathogenesis of NASH. Trends Endocrinol Metab.

[9] Wang S, Sheng F, Zou L, Xiao J, Li P (2021). Hyperoside attenuates non-alcoholic fatty liver disease in rats via cholesterol metabolism and bile acid metabolism. J Adv Res.

[10] Sozen E, Ozer NK (2017). Impact of high cholesterol and endoplasmic reticulum stress on metabolic diseases: An updated mini-review. Redox Biol.

[11] Sozen E, Demirel-Yalciner T, Sari D, Ozer NK (2022). Cholesterol accumulation in hepatocytes mediates IRE1/p38 branch of endoplasmic reticulum stress to promote nonalcoholic steatohepatitis. Free Radic Biol Med.

[12] Hsu SK, Li CY, Lin IL, Syue WJ, Chen YF, Cheng KC (2021). Inflammation-related pyroptosis, a novel programmed cell death pathway, and its crosstalk with immune therapy in cancer treatment. Theranostics.

[13] Coll RC, Schroder K, Pelegrín P (2022). NLRP3 and pyroptosis blockers for treating inflammatory diseases. Trends Pharmacol Sci.

[14] Guo M, Wang X, Zhao Y, Yang Q, Ding H, Dong Q (2018). Ketogenic Diet Improves Brain Ischemic Tolerance and Inhibits NLRP3 Inflammasome Activation by Preventing Drp1-Mediated Mitochondrial Fission and Endoplasmic Reticulum Stress. Front Mol Neurosci.

[15] Lu X, Huang H, Fu X, Chen C, Liu H, Wang H (2022). The Role of Endoplasmic Reticulum Stress and NLRP3 Inflammasome in Liver Disorders. Int J Mol Sci.

[16] Latif MU, Schmidt GE, Mercan S, Rahman R, Gibhardt CS, Stejerean-Todoran I (2022). NFATc1 signaling drives chronic ER stress responses to promote NAFLD progression. Gut.

[17] Bhowmick S, Biswas T, Ahmed M, Roy D, Mondal S (2023). Caveolin-1 and lipids: Association and their dualism in oncogenic regulation. Biochim Biophys Acta Rev Cancer.

[18] Zhao Y, Jia X, Yang X, Bai X, Lu Y, Zhu L (2022). Deacetylation of Caveolin-1 by Sirt6 induces autophagy and retards high glucose-stimulated LDL transcytosis and atherosclerosis formation. Metabolism.

[19] Sun X, Ji G, Li P, Li W, Li J, Zhu L (2021). miR-344-5p Modulates Cholesterol-Induced β-Cell Apoptosis and Dysfunction Through Regulating Caveolin-1 Expression. Front Endocrinol (Lausanne).

[20] Crewe C, Chen S, Bu D, Gliniak CM, Wernstedt Asterholm I, Yu XX (2022). Deficient Caveolin-1 Synthesis in Adipocytes Stimulates Systemic Insulin-Independent Glucose Uptake via Extracellular Vesicles. Diabetes.

[21] Gokani S, Bhatt LK (2022). Caveolin-1: A Promising Therapeutic Target for Diverse Diseases. Curr Mol Pharmacol.

[22] Kuppuswamy D, Chinnakkannu P, Reese C, Hoffman S (2021). The Caveolin-1 Scaffolding Domain Peptide Reverses Aging-Associated Deleterious Changes in Multiple Organs. J Pharmacol Exp Ther.

[23] Okada S, Raja SA, Okerblom J, Boddu A, Horikawa Y, Ray S (2019). Deletion of caveolin scaffolding domain alters cancer cell migration. Cell Cycle.

[24] Lu J, Zhang J, Wang Y, Sun Q (2018). Caveolin-1 Scaffolding Domain Peptides Alleviate Liver Fibrosis by Inhibiting TGF-β1/Smad Signaling in Mice. Int J Mol Sci.

[25] Fu D, Wu S, Jiang X, You T, Li Y, Xin J (2023). Caveolin-1 alleviates acetaminophen-induced vascular oxidative stress and inflammation in non-alcoholic fatty liver disease. Free Radic Biol Med.

[26] Geng Y, Faber KN, de Meijer VE, Blokzijl H, Moshage H (2021). How does hepatic lipid accumulation lead to lipotoxicity in non-alcoholic fatty liver disease?. Hepatol Int.

[27] Ray K (2018). NAFLD-HCC: target cholesterol. Nat Rev Gastroenterol Hepatol.

[28] Li M, Chen D, Huang H, Wang J, Wan X, Xu C (2017). Caveolin1 protects against diet induced hepatic lipid accumulation in mice. PLoS One.

[29] Raza S, Rajak S, Upadhyay A, Tewari A, Anthony Sinha R (2021). Current treatment paradigms and emerging therapies for NAFLD/NASH. Front Biosci (Landmark Ed).

[30] Horn CL, Morales AL, Savard C, Farrell GC, Ioannou GN (2022). Role of Cholesterol-Associated Steatohepatitis in the Development of NASH. Hepatol Commun.

[31] Wang F, Gu HM, Zhang DW (2014). Caveolin-1 and ATP binding cassette transporter A1 and G1-mediated cholesterol efflux. Cardiovasc Hematol Disord Drug Targets.

[32] Sun L, Cai J, Gonzalez FJ (2021). The role of farnesoid X receptor in metabolic diseases, and gastrointestinal and liver cancer. Nat Rev Gastroenterol Hepatol.

[33] Byun S, Jung H, Chen J, Kim YC, Kim DH, Kong B (2019). Phosphorylation of hepatic farnesoid X receptor by FGF19 signaling-activated Src maintains cholesterol levels and protects from atherosclerosis. J Biol Chem.

[34] Adams CJ, Kopp MC, Larburu N, Nowak PR, Ali MMU (2019). Structure and Molecular Mechanism of ER Stress Signaling by the Unfolded Protein Response Signal Activator IRE1. Front Mol Biosci.

[35] Moncan M, Mnich K, Blomme A, Almanza A, Samali A, Gorman AM (2021). Regulation of lipid metabolism by the unfolded protein response. J Cell Mol Med.

[36] Coleman OI, Haller D (2019). ER Stress and the UPR in Shaping Intestinal Tissue Homeostasis and Immunity. Front Immunol.

[37] Celik C, Lee SYT, Yap WS, Thibault G (2023). Endoplasmic reticulum stress and lipids in health and diseases. Prog Lipid Res.

[38] Díaz MI, Díaz P, Bennett JC, Urra H, Ortiz R, Orellana PC (2020). Caveolin-1 suppresses tumor formation through the inhibition of the unfolded protein response. Cell Death Dis.

[39] Zeng W, Tang J, Li H, Xu H, Lu H, Peng H (2018). Caveolin-1 deficiency protects pancreatic β cells against palmitate-induced dysfunction and apoptosis. Cell Signal.

[40] Broz P, Pelegrín P, Shao F (2020). The gasdermins, a protein family executing cell death and inflammation. Nat Rev Immunol.

[41] Le X, Mu J, Peng W, Tang J, Xiang Q, Tian S (2020). DNA methylation downregulated ZDHHC1 suppresses tumor growth by altering cellular metabolism and inducing oxidative/ER stress-mediated apoptosis and pyroptosis. Theranostics.

[42] de la Roche M, Hamilton C, Mortensen R, Jeyaprakash AA, Ghosh S, Anand PK (2018). Trafficking of cholesterol to the ER is required for NLRP3 inflammasome activation. J Cell Biol.

[43] Drummer C 4th, Saaoud F, Jhala NC, Cueto R, Sun Y, Xu K (2023). Caspase-11 promotes high-fat diet-induced NAFLD by increasing glycolysis, OXPHOS, and pyroptosis in macrophages. Front Immunol.

[44] Gaul S, Leszczynska A, Alegre F, Kaufmann B, Johnson CD, Adams LA (2021). Hepatocyte pyroptosis and release of inflammasome particles induce stellate cell activation and liver fibrosis. J Hepatol.

[45] Jiang X, Li Y, Fu D, You T, Wu S, Xin J (2023). Caveolin-1 ameliorates acetaminophen-aggravated inflammatory damage and lipid deposition in non-alcoholic fatty liver disease via the ROS/TXNIP/NLRP3 pathway. Int Immunopharmacol.

[46] Lu J, Zhang J, Wang Y, Sun Q (2018). Caveolin-1 Scaffolding Domain Peptides Alleviate Liver Fibrosis by Inhibiting TGF-β1/Smad Signaling in Mice. Int J Mol Sci.

[47] Lim JE, Bernatchez P, Nabi IR (2024). Scaffolds and the scaffolding domain: an alternative paradigm for caveolin-1 signaling. Biochem Soc Trans.

[48] Marudamuthu AS, Bhandary YP, Fan L, Radhakrishnan V, MacKenzie B, Maier E (2019). Caveolin-1-derived peptide limits development of pulmonary fibrosis. Sci Transl Med.

[49] Mundhara N, Majumder A, Panda D (2019). Methyl-β-cyclodextrin, an actin depolymerizer augments the antiproliferative potential of microtubule-targeting agents. Sci Rep.

[50] El Kasmi KC, Ghosh S, Anderson AL, Devereaux MW, Balasubramaniyan N, D'Alessandro A (2022). Pharmacologic activation of hepatic farnesoid X receptor prevents parenteral nutrition-associated cholestasis in mice. Hepatology.

[51] Liu J, Li Y, Sun C, Liu S, Yan Y, Pan H (2020). Geniposide reduces cholesterol accumulation and increases its excretion by regulating the FXR-mediated liver-gut crosstalk of bile acids. Pharmacol Res.

