# Supplementary material for: Caveolin-1 mitigates the advancement of metabolic dysfunction-associated steatotic liver disease by reducing endoplasmic reticulum stress and pyroptosis through the restoration of cholesterol homeostasis
Source: Int J Biol Sci. 2025 Jan 1;21(2):490–506. doi: 10.7150/ijbs.100794 (PMC11705642; doi:10.7150/ijbs.100794)
Supplement: Supplementary file 1 — Supplementary tables. [file ijbsv21p0490s1.pdf]

Supplementary information

Table S1. The characteristics of human subjects

NO.	age	gender	Ethnicity	Disease diagnosis	Liver characteristics
1	73	Man	Chinese	hepatocellular carcinoma	Normal (Pericancerous tissue)
2	49	Man	Chinese	liver neoplasms	Normal (Pericancerous tissue)
3	35	Woman	Chinese	Hepatic hemangioma	Normal
4	68	Man	Chinese	Hepatocellular adenoma	Normal (Pericancerous tissue)
5	28	Man	Chinese	Chronic hepatitis B	Masld
6	36	Man	Chinese	Chronic hepatitis B	Masld
7	35	Man	Chinese	Chronic hepatitis B	Masld
8	49	Man	Chinese	Severe fatty liver disease	Masld
9	56	Woman	Chinese	Polycystic disease in adults with fatty liver	Masld
10	33	Woman	Chinese	Gallstones with cholecystitis	Masld
11	65	Woman	Chinese	Chronic cholecystitis with cholesterol polyps	Masld
12	59	Woman	Chinese	Chronic hepatitis	Masld
13	42	Man	Chinese	Chronic hepatitis B	Fibrosis
14	28	Man	Chinese	Chronic hepatitis B	Fibrosis
15	37	Woman	Chinese	Chronic hepatitis	Fibrosis
16	50	Woman	Chinese	Gallbladder rupture	Fibrosis

Table S2. List of antibodies used in this study.

Antibody	Source	Application
CAV1	ProteinTech	WB: 1:3000 IHC: 1:5000
Vimentin	ProteinTech	WB: 1:2000
α-SMA	Abmart	WB: 1:1000
CollagenI	Abcam	WB: 1:1000
ABCG5	Affinity	WB: 1:1000 IHC: 1:200
ABCG8	Affinity	WB: 1:1000
NR1H4	ProteinTech	WB: 1:1000 IHC: 1:200 IF: 1:200
SREBP1	ProteinTech	WB: 1:1000
PPARγ	Affinity	WB: 1:1000
PPARα	Affinity	WB: 1:1000
GRP78	Abcam	WB: 1:1000 IHC: 1:100 IF: 1:100
CHOP	Cell Signaling	WB: 1:2000 IF: 1:150
p-PERK	Cell Signaling	WB: 1:1000
PERK	Cell Signaling	WB: 1:2000
p-EIF2α	Cell Signaling	WB: 1:1000
EIF2α	Cell Signaling	WB: 1:2000
NLRP3	Affinity	WB: 1:1000 IHC: 1:100 IF: 1:100
IL-1β	Affinity	WB: 1:2000
GSDMD-N	HuaBio	WB: 1:1000 IHC: 1:200
Caspase1-p20	Affinity	WB: 1:1000
β-actin	HuaBio	WB: 1:5000

Table S3. List of primers used in this study.

Name	Sequence (5’-3’)	Species
ABCG5 Forward	TGGATCCAACACCTCTATGCTAAA	Mouse
Reverse	GGCAGGTTTTCTCGATGAACTG	
ABCG8 Forward	GTAGCTGATGCCGATGACAA	Mouse
Reverse	GGGGCTGATGCAGATTCA	
NR1H4 Forward	GCTTGATGTGCTACAAAAGCTG	Mouse
Reverse	CGTGGTGATGGTTGAATGTCC	
GRP78 Forward	ACATGGACCTGTTCCGCTCTA	Mouse
Reverse	TGGCTCCTTGCCATTGAAGA	
GRP94 Forward	TTGTGTCCAATTCAAGGTAATCA	Mouse
Reverse	TTGCTGACCCAAGAGGAAAC	
CHOP Forward	CCACCACACCTGAAAGCAGAA	Mouse
Reverse	GGTGCCCCCAATTTCATCT	
β-actin Forward	CTCTTCCAGCCTTCCTTCCT	Mouse
Reverse	AGCACTGTGTTGGCGTACAG	
NLRP3 Forward	ATTACCCGCCCGAGAAAGG	Mouse
Reverse	TCGCAGCAAAGATCCACACAG	
IL-1β Forward	GCAGGCAGTATCACTCATTGTGG	Mouse
Reverse	GAGTCACAGAGGATGGGCTCTTC	
IL-18 Forward	GACTCTTGCGTCAACTTCAAGG	Mouse
Reverse	CAGGCTGTCTTTTGTCAACGA	
